# Differences in Behavior and Activity Associated with a Poly(A) Expansion in the Dopamine Transporter in Belgian Malinois

**DOI:** 10.1371/journal.pone.0082948

**Published:** 2013-12-23

**Authors:** Lisa Lit, Janelle M. Belanger, Debby Boehm, Nathan Lybarger, Anita M. Oberbauer

**Affiliations:** 1 Department of Animal Science, University of California Davis, Davis, California, United States of America; 2 Precision Canine, Phoenix, Arizona, United States of America; 3 Left Coast K9, Marysville, California, United States of America; Osaka University Graduate School of Medicine, Japan

## Abstract

In Belgian Malinois dogs, a 38-base pair variable number tandem repeat in the dopamine transporter gene (*SLC6A3*) is associated with behavior changes in Malinois. By additional sequencing in *SLC6A3*, we identified an intronic 12-nucleotide poly(A) insertion (“PolyA(22)”) before the terminal exon that was associated with seizure, “glazing over” behaviors, and episodic biting behaviors in a sample of 138 Malinois. We next investigated whether PolyA(22) was associated with 1) increased locomotor activity and 2) response to novelty. Using a sample of 22 Malinois and 25 dogs of other breeds, dogs’ activity was monitored in a novel and non-novel environment while wearing activity monitoring collars. All dogs were more active in novel compared with non-novel environments, and Malinois were more active overall than other breeds. There was an effect of PolyA(22) genotype on activity levels, and this effect appeared to underlie the difference detected between Malinois and other breeds. There was no effect of PolyA(22) genotype on the relative decrease in activity between novel and non-novel environments for either group or all dogs considered together. In addition to an association between PolyA(22) and owner reports of seizure, “glazing over” behaviors, and episodic biting behaviors, these findings support an effect of PolyA(22) on dopamine transporter function related to activity. Further investigation is required to confirm mechanistic effects of PolyA(22) on *SLC6A3*. The complex polygenic nature of behavior and the range of behaviors associated with this insertion predict that effects are likely also modified by additional genetic and environmental factors.

## Introduction

The neurotransmitter dopamine has been implicated in pathogenesis of a wide range of behaviors, including aggression, in part due to its wide-ranging role as a neuromodulator (reviewed in [1]). Dopaminergic involvement in aggressive behavior is further supported by pharmacotherapeutic intervention with dopaminergic antagonists [2], [3]. However, the complex polygenic nature of aggression suggests that the same genetic polymorphism combined with different environmental or genetic backgrounds may result in a wide array of expressions of behavior, including the display of aggression associated with that polymorphism (e.g. [4]).

Because aggression in dogs is considered a serious threat to public health [5], identification of genetic polymorphisms associated with aggressive behavior may be helpful to dog breeders and owners. A dopaminergic gene that is relevant to aggression and other behavioral changes across species is the dopamine transporter (*SLC6A3*). *SLC6A3* encodes a protein responsible for regulation of signal amplitude and duration in dopaminergic synapses [6]. In humans *SLC6A3* has been associated with aggression [7], hyperactivity [8], and response to novelty [9]. Findings in SLC6A3 knockout mice of differences in aggressive and other social behaviors [10], hyper-locomotion [10], and response to a novel environment [11] further demonstrate a mechanistic link between *SLC6A3* and a range of behavioral phenotypes.

In dogs, a variable number tandem repeat (VNTR) in *SLC6A3* is over-represented in Belgian Malinois (“Malinois”) in Europe and the United States [12], [13] and has been associated with stress-related behaviors in Belgian military dogs [12], including dogs’ yawning, lowered posture, and hyper-vigilance to handlers, as well as increased handlers’ use of aversive stimuli in some environments. Increased dog attentiveness to their handlers and episodic behavioral changes associated with this VNTR was also reported by owners of Malinois in the United States [12]. Deep sequencing across *SLC6A3* revealed substantial genetic heterogeneity across *SLC6A3* in Malinois that was not previously identified in other breeds [14].

Here we report on two novel alleles identified through additional fine mapping: an 18-nucleotide deletion found in most breeds we evaluated, and a 12-nucleotide poly(A) insertion found primarily in Belgian Malinois [“PolyA(22)”] that, like the VNTR previously described [12] is associated with owner reports of episodic behavioral changes in dogs, but we hypothesize may be more likely to result in mechanistic changes to *SLC6A3* than the VNTR previously described.

Identification of predictive behavior measures associated with a specific genetic polymorphism can assist in understanding the broader nature of *SLC6A3*-related behavioral phenotypes in dogs. In particular, it is difficult to assess sporadic behavioral outbursts with tests designed to provoke spontaneous aggression as these can be risky to assess. Therefore it is important to identify quantifiable changes in relevant behavior that can be evaluated with minimal risk to owners and evaluators. Although our previous study utilized execution of specific trained tasks in working dogs [12], protocols that do not require specific training prior to assessment are more practical to assess a broader sample of dogs. Because we hypothesized that PolyA(22) would impact dopaminergic function, additional effects on locomotion and/or response to novelty were possible. This hypothesis was partly confirmed: activity levels were associated with PolyA(22), with no relationship between PolyA(22) and response to novelty in our subjects.

## Materials and Methods

### Ethics Statement

All samples were collected in accordance with protocol approval by the Animal Care and Use Committee at the University of California at Davis. Owners of the dogs gave permission for their animals to be used in this study.

### Subjects

Malinois and other breeds (“Other”) were recruited through word of mouth to provide blood and/or buccal swab samples for DNA analysis. For genotyping purposes, additional samples from Malinois and Other were obtained from the Canine Genetic Analysis Project (CGAP) sample base (http://cgap.ucdavis.edu/). For the activity study, dogs were required to be between five months and 10 years of age, and in good health with no health or movement problems. Owners were requested to provide dog age, sex, breed, and intact status.

### DNA Collection, Amplification, and Genotyping

Buccal derived DNA was collected by owners, then extracted and purified using previously described methods [15]. The DNA was amplified with primers that flanked chr34∶11243777–11244026 (CanFam 3.1) (forward: FAM-labeled 5′CAGATCAGACATTACTCTAACTATTGC and reverse: 5′TCATCAAGCAGGGAAAAAGG). The total volume of the PCR reaction was 20 µl. For each PCR reaction, 1 µl of buccal swab DNA was used. A master mix with final PCR reaction for each sample contained 1X Applied Biosystems taq polymerase buffer II (Applied Biosystems, Carlsbad, CA), 1.5 mM MgCl2 (Applied Biosystems), 200 µM dNTPs (Promega, Madison, WI), 1 unit of Amplitaq DNA polymerase (Applied Biosystems) and 0.2 µM of each forward and reverse primer (Fisher Scientific). An MJ Research PTC-200 thermal cycler (MJ Research, Inc., Incline Village, NV) was used for DNA amplification. Samples were heated to 95°C for 5 minutes for initial denaturation, followed by 35 cycles of 30 sec at 95°C, 30 sec at 60°C, 30 sec at 72°C, and a 10 minute final extension at 72°C. PCR products (one microliter) were genotyped using ABI 3100 Capillary Electrophoresis Genetic Analyzer at the UC Davis DNA Sequencing Facility (http://dnaseq.ucdavis.edu) and analyzed using STRand Analysis Software (http://www.vgl.ucdavis.edu/STRand) [16].

### Associated Behaviors in Malinois

To investigate whether the PolyA(22) allele was associated with owner reports of changes in behaviors, owners of Malinois were asked whether their dogs had ever had 1) seizures; 2) eyes glazing over and loss of responsiveness to environmental stimuli; or 3) sudden brief episodes of aggressive displays with no apparent trigger, directed towards one or more of the following: the owner, other people, or other dogs. Owner responses were coded dichotomously (Yes, No) for any affirmative response to questions regarding dog change in behavior as described above. Seizures were owner-reported and not necessarily verified by veterinarian observation, due to their episodic occurrence. Behavior data for each dog included was verified by experimenters familiar with individual dogs. Many of the participants with Malinois obtained their dogs either through importers of working canines or rescue organizations. Because of the difficulty in documenting the origin and actual age of many of the dogs utilized by law enforcement agencies and dogs obtained through Malinois rescue organizations, data were analyzed without considering effects of age or familial relationships. A pedigree drawing for Malinois with known background illustrates that the dataset consists of dogs across a wide range of backgrounds through multiple generations (Figure S1; gold boxes indicate dogs in our dataset).

### Activity Monitoring

Evaluations were conducted at the Cognitive Canine Research Center at the University of California Davis (CCRC) and at the Precision Canine Training Center in Phoenix, Arizona (PC). Dog activity was evaluated in a room (EVAL_ROOM: approximately 6 meters × 6 meters) that was initially novel to all dogs. Dogs entered EVAL_ROOM with their owners and were allowed to freely explore the space for five minutes (Time 1, T1). During this time, the owner was seated in a chair provided on one side of the room; one experimenter, novel to all dogs, was standing against the wall across and at the opposite end of the room from the owner; an additional experimenter with a video camera was located behind a baby gate. An empty wire dog crate (closed and latched) was located across from the owner. Owners were instructed to minimize physical contact and verbal interaction with their dogs. Pilot studies showed that most dogs were significantly inhibited when owners and experimenters remained silent during data collection (data not shown). Thus for this study, owners and experimenters maintained normal conversation during all data collection periods. Dogs were then taken by their owner to a second adjacent location for five minutes, accompanied by both experimenters, who remained standing; and owners, who sat in a chair while dogs freely explored this area. Owners, dogs, and experimenters then returned to EVAL_ROOM and repeated the procedure described above for five minutes (Time 2, T2).

Prior to entering EVAL_ROOM the first time, dogs were fitted with a collar containing an Actical activity monitoring unit (http://www.minimitter.com/actical_animal.cfm) set to record activity at 15-second epochs. Dependent variables recorded were activity in EVAL_ROOM for T1 (T1-ACTIV) and T2 (T2-ACTIV) in EVAL_ROOM, and total activity T1+T2 (TOT-ACTIV).

### Statistical Analyses

Data were analyzed using IBM SPSS Version 21 [17]. All analyses used a significance threshold of α <0.05 (two-tailed).

Genotype frequencies and responses to owner report of behavior questions were analyzed using either a *χ* ^2^ test of independence or goodness of fit test, as appropriate.

Komolgorov-Smirnov *Z* Tests were used to evaluate normal distributions for age at testing, T1-ACTIV, T2-ACTIV, and TOT-ACTIV. Independent samples *t*-tests and one-way ANOVA were used to evaluate differences between locations (CCRC and PC) for T1-ACTIV, T2-ACTIV, and TOT-ACTIV, and differences in age between Malinois and Other and across PolyA(22) genotypes (0/0: No PolyA(22) alleles; 0/PolyA(22): one PolyA(22) allele; PolyA(22)/PolyA(22): two PolyA(22) alleles). Chi-square tests of independence were used to determine differences in neuter status across sex, and differences in sex and neuter status between Malinois and Other. One-way ANOVA was used to evaluate effects of sex and neuter status on T1-ACTIV, T2-ACTIV, and TOT-ACTIV within Malinois and Other. To consider effects Breed on differences in activity levels, a mixed between-groups (Breed: Malinois, Other) within-group (Time: T1, T2) 2×2 ANOVA was used. To consider effects of PolyA(22) on activity level differences, a mixed between-groups (Genotype: 0/0, 0/PolyA(22), PolyA(22)/PolyA(22)) within-group (Time: T1, T2) 3×2 ANOVA was used. Bonferroni corrections were used in pairwise comparisons to correct for multiple comparisons. Partial eta squared (*η* ^2^) is provided as a measure of effect size. An exploratory standard multiple regression was used to examine relationships between PolyA(22) genotype, sex, neuter status, location, and age at testing. Video recording was not evaluated for this study.

## Results

### *SLC6A3* Sequencing

**Figure 1:**
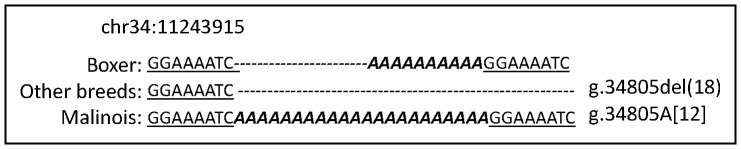
Three alleles at polyA site. Allele differences beginning at chr34∶11243915 (Broad CanFam3.1) for Boxer reference sequence (“polyA(10)”), the allele most common in other breeds in our dataset (g.34805del(18); “polyA(0)”), and the allele (g.34805A(12); “PolyA(22)”) found primarily in Malinois in our dataset.

Using 20 Malinois previously selected for *SLC6A3* sequencing [14], we determined that intronic SNP ss709035061 (chr34∶11242320) was significantly associated with owner report of changes in behavior as described above (*Χ* ^2^ = 18.398, *p* = 1.79E-05). Further sequence analysis identified a 22-bp intronic poly(A) expansion in Malinois at g.34797 (chr34∶11243915, Broad CanFam3.1) 5′ of the terminal exon. We genotyped this insertion in 234 dogs (165 Malinois, 69 Other; Table S1), identifying three alleles. In our sample the rarest allele was reflected by the Boxer reference sequence (Broad CanFam3.1): a 10-nucleotide poly(A) sequence flanked by an 8-bp target site duplication (GGAAAATC) [“PolyA(10)”] (Figure 1). The common sequence in dogs other than Malinois consisted of only the left 8-bp sequence with no poly(A) sequence and no right flanking 8-bp sequence (g.34805del(18)) [“PolyA(0)”] (Figure 1). Finally, found primarily in Malinois, an additional allele was identified with the same 8-bp target site duplication seen in Boxers and a 22-bp poly(A) sequence (g.34805A [12]) [“PolyA(22)”] (Figure 1). The relationship between the previously reported *SLC6A3* VNTR and the poly(A) expansion is provided in Table S1.

### PolyA(22) Association with Owner-reported Behavior in Malinois

**Figure 2:**
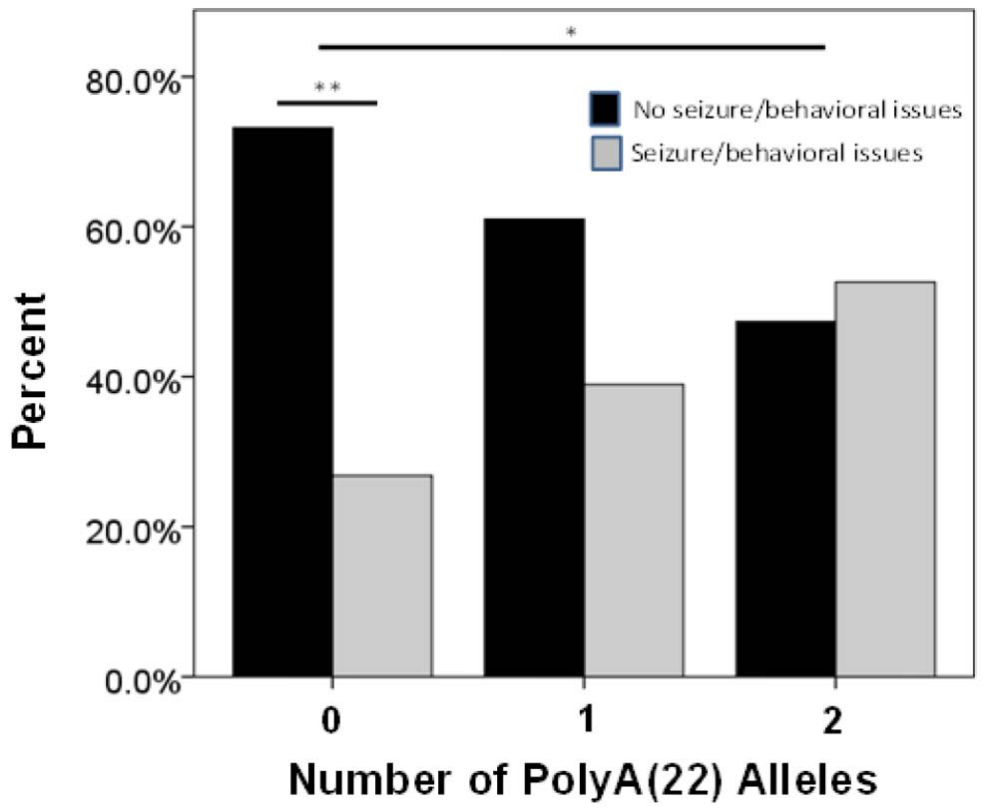
PolyA alleles and owner-reported behavioral changes. General behavior changes: Distribution for number of PolyA(22) alleles (0: Malinois with no PolyA(22) alleles; 1: Malinois with one PolyA(22) allele; 2: Malinois homozygous for PolyA(22)) for owners reporting seizures or unpredictable behavioral changes including dogs’ eyes “glazing over”, dogs’ lack of response to environmental stimuli, and loss of behavioral inhibition (gray bars) or no seizure or behavioral issues (black bars). *: *p*<0.05; **: *p*<0.01.

**Figure 3:**
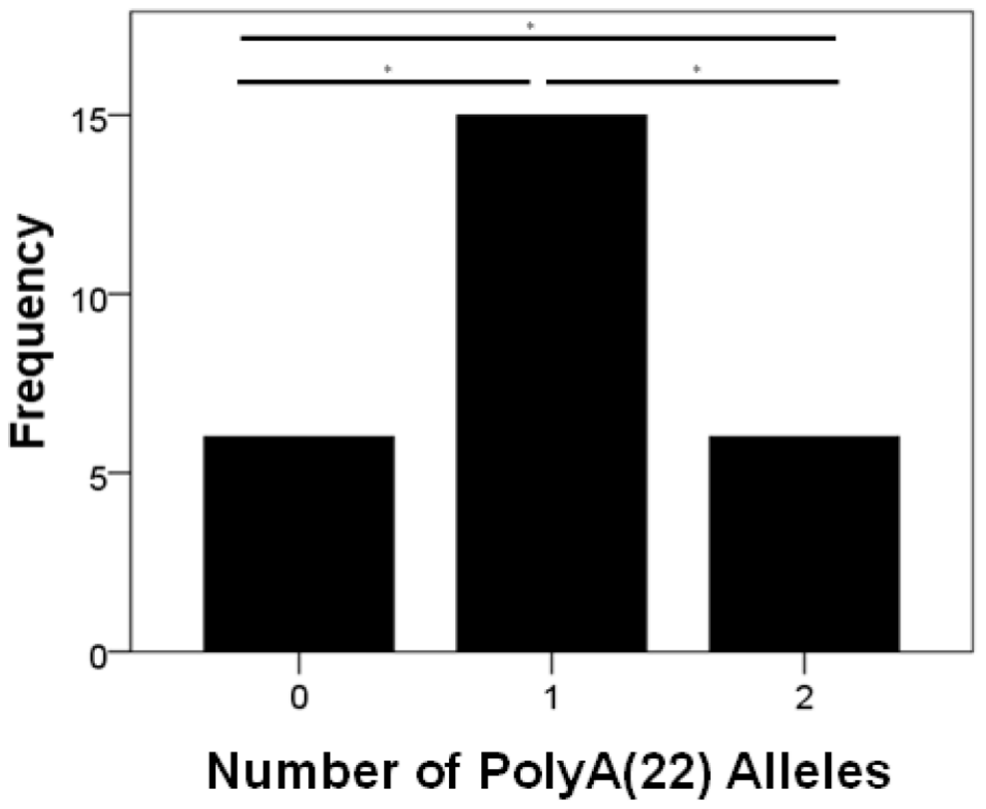
PolyA alleles and owner-reported loss of responsiveness to environmental stimuli (“glazing over”). Glazing over: Distribution for number of PolyA(22) alleles (0: Malinois with no PolyA(22) alleles; 1: Malinois with one PolyA(22) allele; 2: Malinois homozygous for PolyA(22)) for owners only reporting dogs’ eyes “glazing over”. *: *p*<0.05.

When considering owner report of changes in behavior in Malinois (*n* = 138), there was no effect of dog sex on owner responses to questions (Table S2). Owners of Malinois with two PolyA(22) alleles were more likely to report at least one of the behavioral issues than owners of Malinois with no PolyA(22) alleles (*χ^ 2^*(1) = 5.5, *p* = 0.02, *Φ* = 0.26); owners of Malinois with no PolyA(22) alleles were more likely to report no issues (*χ^ 2^*(1) = 8.9, *p* = 0.003, *Φ* = 0.21), while reports of issues for Malinois with at least one PolyA(22) allele were intermediate between dogs homozygous for PolyA(22) and dogs with no copies of this allele (Figure 2). For owners reporting dogs only displaying glazing over (*n* = 27), dogs were significantly more likely to carry only a single PolyA(22) allele (*χ^ 2^*(1) = 6, *p* = 0.05, *Φ* = 0.11) (Table S3, Figure 3). For owners reporting dogs displaying only episodic aggression (*n* = 38), dogs were more likely to carry at least one PolyA(22) allele (*χ^ 2^*(1) = 17.79, *p*<0.001, *Φ* = 0.47) (Table S3). This was also true for owners reporting dogs who both glazed over and displayed episodic aggression (*n* = 15); all but one of these dogs had at least one PolyA(22) allele (*χ^ 2^*(1) = 11.27, *p* = 0.001, *Φ* = 0.75) (Table S3).

To confirm that owner reports of changes in behavior were not limited to a single family subunit, a pedigree chart was generated coloring nodes for Malinois with pedigree information available and whose owners reported at least one behavioral issue. This chart illustrates that behavioral issues were broadly distributed throughout and not restricted to familial subunits (Figure S2).

### Activity Sample Description

Twenty-two Belgian Malinois (Malinois: 64% Male; 73% Intact) and 25 dogs of other breeds (“Other”: 24% Male; 20% Intact), participated in this study (Table 1). Age information was provided for 20 Malinois and 16 Other (Malinois: M = 37.5 months, SD = 20.3 months; Other: M = 53.7 months, SD = 29.7 months). Age at testing, T1-ACTIV and T2-ACTIV were normally distributed (K-S *Z*, all *p*>0.05). Overall PolyA(22) genotypes were determined for 39 dogs (22 Malinois, 17 Other) (Table 2). As the reference sequence was the least represented, subjects with common and reference genotypes were combined for analysis (Table 2). Distribution of PolyA(22) genotypes was different between Malinois and Other [*χ* ^2^(2) = 19.9, *p*<0.001, *Φ* = 0.71]; more Malinois had at least one polyA(22) allele than Other ([*χ* ^2^(1) = 19.7, *p*<0.001, *Φ* = 0.71] (Table 2).

**Table 1:** Breed Distributions for dogs included in activity study.

Breed	*n*	(%)	% Male	% Neutered
Malinois	22	(100.0)	63	27.3
**Other Breeds:**				
Border Collie	2	(8.0)		
Boxer	2	(8.0)		
Belgian Sheepdog	1	(4.0)		
Cocker Spaniel	1	(4.0)		
German Shepherd	3	(12.0)		
Labrador Retriever	1	(4.0)		
Mixed Breed	3	(12.0)		
Pit Bull Terrier	4	(16.0)		
Rottweiler	1	(4.0)		
Silkie Terrier	3	(12.0)		
Springer Spaniel	2	(8.0)		
Weimeraner	2	(8.0)		
**Total Other Breeds**	25	(100.0)	24	76

**Table 2:** PolyA(22) genotype by Malinois/Other for dogs in activity study.

Breed	Genotype	*n* (%)
Malinois	No PolyA(22) Alleles	5 (22.7)
	One PolyA(22) Allele	8 (36.4)
	Two PolyA(22) Alleles	9 (40.9)
	**Total**	22 (100.0)
Other Breeds	No PolyA(22) Alleles	16 (94.1)
	One PolyA(22) Allele	1 (5.9)
	**Total**	17 (100.0)

### Effect of Age, Location, Sex, and Neuter Status

There was no significant difference in age (months) between Malinois and Other [*t*(25.5) = −1.87, *p* = 0.07, equal variance not assumed], or age across PolyA(22) genotypes [*F*(2, 28) = 1.24, *p* = 0.31]. Therefore age was not utilized as a covariate in the following analyses, with the exception of the exploratory regression analysis. There was no significant effect of location (CCRC: *n* = 11, PC: *n* = 36) on T1-ACTIV, T2-ACTIV, or TOT-ACTIV (*t*-test, all *p*>0.05), and therefore location was not considered in the following analyses, with the exception of the exploratory regression analysis. There was no significant difference in Neuter Status (Intact, Neutered) across Sex (Male, Female) (*χ* ^2^, *p* = 0.5). Compared with Other, more Malinois were Male [*χ* ^2^(1) = 7.52, *p* = 0.006] and Intact [*χ* ^2^(1) = 11.75, *p* = 0.001]. However within Malinois and Other considered separately, there was no effect of Sex, Neuter Status, or Sex*Neuter Status on T1-ACTIV, T2-ACTIV, or TOT-ACTIV (ANOVA, all *p*>0.05), and therefore Sex and Neuter Status were not utilized as covariates in the following analyses, with the exception of the exploratory regression analysis.

### Effect of Malinois vs. Other on Overall Activity

For the omnibus 2×2 [Time (T1, T2) × Breed (Malinois, Other)] ANOVA, there was a main effect of Time [F(1,45) = 21.41, *p*<0.001, *η* ^2^ = 0.32]; dogs were more active the first time in the testing room than the second time. There was a main effect of Breed [F(1,45) = 7.59, *p* = 0.008, *η* ^2^ = 0.14]; Malinois were more active than Other. There was no interaction between Time and Breed (*p* = 0.59).

### Effect of PolyA(22) Genotype for Malinois and Other Considered Separately

Because the genotypes 0/PolyA(22) and PolyA(22)/PolyA(22) are more common in Malinois than Other, it was possible that differences in genotype may contribute to observed differences in activity. In the current sample, the Other group of dogs predominantly were genotype 0/0 (0/0: *n* = 16; 0/PolyA(22): *n* = 1; PolyA(22)/PolyA(22): *n* = 0, *χ* ^2^<0.001, Cohen’s *w* = 0.88) (Table 2); therefore effect of PolyA(22) genotype could not be determined for that group separately.

Within the Malinois group, for the 2×3 [Time (T1, T2) × PolyA(22) Genotype (0/0, 0/PolyA(22), PolyA(22)/PolyA(22))] ANOVA, there was a main effect of Time [F(1, 19) = 4.98, *p* = 0.04, *η* ^2^ = 0.21]; dogs were more active the first time in the testing room than the second time. There was no main effect of PolyA(22) genotype [*p* = 0.17], and no interaction between Room and PolyA(22) genotype (*p* = 0.56). Only five Malinois were PolyA(22) genotype 0/0 (Table 2), and the resulting power (*β* = 0.35) may have been insufficient to identify between-group differences.

### Effect of PolyA(22) Genotype for Malinois and Other Considered Together

To determine whether Malinois and Other dogs with no PolyA(22) alleles could be combined for analysis, activity levels for these dogs were compared across Malinois and Other. For dogs with genotype 0/0, there was no difference between Malinois and Other in TOTAL_ACTIV, T1-ACTIV or T2-ACTIV [*t*(19), all *p*>0.05]. Therefore Malinois and Other were combined for purposes of examining effects of PolyA(22) genotype.

**Figure 4:**
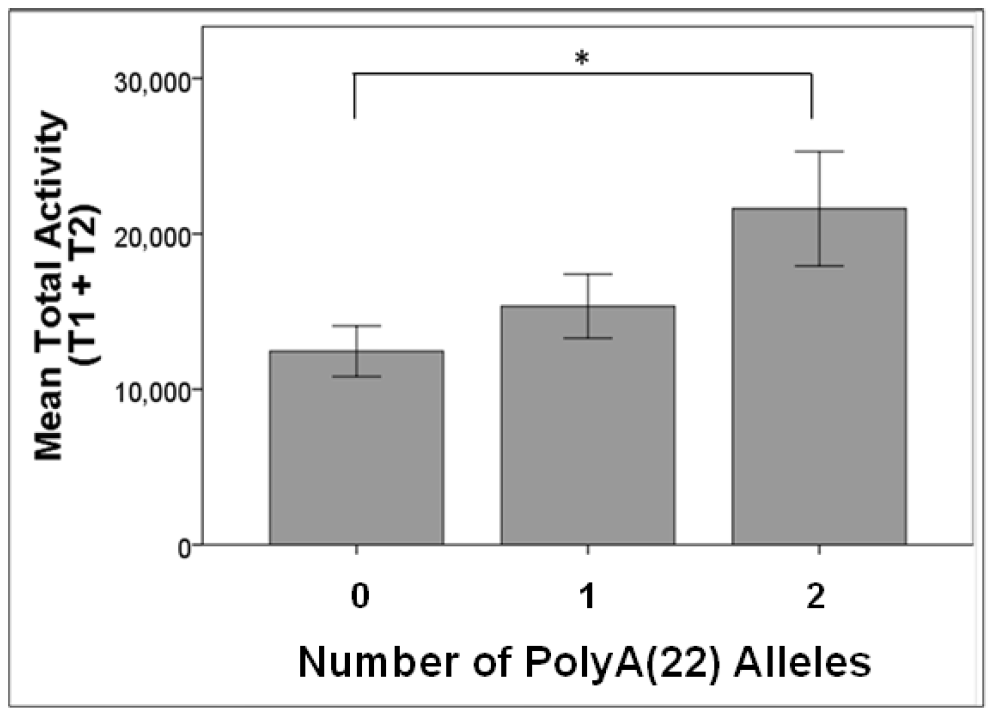
PolyA alleles and activity differences. Activity differences: Mean total activity for number of PolyA(22) alleles (0: dogs with no PolyA(22) alleles; 1: dogs with one PolyA(22) allele; 2: dogs homozygous for PolyA(22)) (Malinois and other breeds combined) (*p<0.05).

With all dogs, for the 2×3 [Time (T1, T2) × PolyA(22) genotype (No Alleles, 1 Allele, 2 Alleles)] ANOVA, there was a main effect of Time [F(1,36) = 12.95, *p* = 0.001, *η* ^2^ = 0.27]; dogs were more active the first time in the testing room than the second time. With the resulting increased power (*β* = 0.68), there was a main effect of PolyA(22) genotype [F(1,36) = 3.98, *p* = 0.02, *η* ^2^ = 0.18]. When considering pairwise comparisons between genotypes, dogs with genotype PolyA(22)/PolyA(22) were more active than dogs with genotype 0/0 (*p* = 0.023, Bonferroni-adjusted); dogs with genotype 0/PolyA(22) were intermediate between dogs with genotypes 0/0 and PolyA(22)/PolyA(22) (Figure 4). Although there was no interaction between Room and PolyA(22) genotype (*p* = 0.64), post-hoc comparisons found that dogs with genotype PolyA(22)/PolyA(22) were significantly more active than dogs with genotype 0/0 for both T1 and T2 (T1: *p* = 0.03, T2: *p* = 0.05, Bonferroni-adjusted).

### Exploratory Regression Analysis

To confirm these findings, an exploratory standard multiple regression analysis was performed between TOTAL_ACTIV as a dependent variable and sex, neuter status, age at testing, location, and PolyA(22) genotype as independent variables (Table 3). The only significant contribution to the TOTAL_ACTIV regression model was made by PolyA(22) genotype (*p* = 0.02; 95% confidence interval 747.8 to 8196.0). Although the bivariate correlation between age at testing and TOTAL_ACTIV was significant (*p* = 0.03; Table 3), age at testing did not contribute significantly to the regression model, suggesting that the relationship between TOTAL_ACTIV and age at testing was mediated by the relationship between PolyA(22) genotype and TOTAL_ACTIV.

**Table 3:** Correlations and standard multiple regression of variables on dependent variable (DV) Total Activity.

Variables	Total Activity (DV)	Sex	Neuter Status	Age at Testing	Location	B	β
Sex	−.280					−2320.337	−0.135
Neuter	−.222	.225				1412.573	0.082
Age at Testing	−.336*	.117	.486**			−88.962	−0.272
Location	−.037	.099	−.120	−.240		−3150.710	−0.109
PolyA(22) Genotype	.513**	−.273	−.349	−.259	0.067	4471.903*	0.441
						*R* ^2^ = 0.34*	
						Adjusted *R* ^2^ = 0.21*	
						*R* = 0.58*	

*p*< = 0.05; **p< = 0.01.

## Discussion

In this study, we identified a 12-nucleotide poly(A) expansion present in Malinois that is associated with owner reports of seizure, episodic behavior changes, and loss of responsiveness to environmental stimuli. In Boxers and Malinois, the sequence is representative of a poly(A) retrotransposon, where a poly(A) expansion is flanked by an identical sequence. Interestingly, in our sample, the reference sequence was found only in the two Boxers assessed. Breeds other than Malinois primarily displayed a third variant that was an 18-nucleotide deletion compared with the reference sequence.

In addition, we demonstrated that dogs were more active in a novel versus non-novel environment independent of PolyA(22) genotype, but that dogs with PolyA(22) alleles were more active overall. The Malinois is typically considered a high-energy breed, requiring ample opportunity for active exercise [18], but we found no difference in activity levels when comparing activity of Malinois with no PolyA(22) alleles with other breeds with no PolyA(22) alleles. Thus, the characterization of the Malinois breed as high-energy may arise in part because the PolyA(22) allele is common within the Malinois breed, and the increased activity levels are associated with genotypes containing one or two PolyA(22) alleles.

Our findings suggest that one functional effect associated with PolyA(22) is increased locomotor activity. This is corroborated by studies in *SCLA3* knockout mice and mice with reduced expression of *SLC6A3* [10], [19], [20]. Both knockout mice and mice with reduced SLC6A3 expression displayed increased locomotor activity [19], [20]. Taken together, this suggests that PolyA(22) reduces expression of *SLC6A3*.

In contrast to our findings, *SLC6A3* knockout mice demonstrate enhanced response to novelty [19], while mice with reduced *SLC6A3* expression show pronounced decrease in activity upon environmental acclimatization compared with wild-type mice [20]. Because we did not find any differences in relative activity with environmental acclimatization, the response to novel versus non-novel environment may be mediated by factors other than, or in addition to, differential *SLC6A3* expression.

Canine studies utilizing paradigms that require some training prior to evaluation may confound findings arising from factors such as individual dog motivation. Our study utilized a simple paradigm that required no training and provided an objective quantitative measure of activity. The absence of detectable activity differences across breeds for novel versus non-novel environments indicates that the task can be further simplified to only include a single exposure in a novel environment in order to obtain relevant data. It is possible that in dogs, alternative behavior assays might identify distinct responses to novelty that would be sensitive to PolyA(22) genotype or breed, that response to novelty may be sensitive to other *SLC6A3* polymorphisms, or that using other breeds may identify distinct breed tendencies. Although training methodologies and background were not included as variables in our studies, it is possible that training background may affect activity. However, given our prior findings that suggested interactions between stress, training, handling, and dopamine transporter polymorphism phenotypes, large targeted studies may be required to elucidate the contribution of training background to *SLC6A3*-related effects on behavior [12].

It is important to note that behavior is notoriously complex. The same genetic polymorphism can result in a wide range of phenotypic expression. Moreover, behavioral differences arising from a single polymorphism can vary according to other genetic and environmental backgrounds [21]. The domestic dog is widely supported as a model for behavior genetics investigations. However the complex nature of behavior and environmental contribution effects create difficulties in accurately phenotyping relevant behaviors [22], including aggression (e.g. [23], [24]). Therefore, as has been done with rodents and human behavior studies, it is important to recognize pleiotropic effects of genetic polymorphisms and identify relevant behaviors where these polymorphisms contribute significantly to variance in behavior. To effectively capitalize on the increasing genetic findings associated with aggression, large collaborative efforts such as those underway in human psychiatric disorders will allow consolidation of findings across relevant canine genes and breeds. For example, broad studies may allow identification of genetic modifiers that predict targets of aggression (i.e., dog aggression or human aggression (child and/or adult)). Development of relatively simple observational paradigms that require no or minimal training and provide objective quantitative data can provide future studies with effective measures as well as offer clinicians diagnostic options to guide behavioral or pharmacological interventions.

We suggest that this insertion represents a genetic change with a functional effect on these behaviors in at least one breed. This may be due to downstream hindrance of poly(A) tail post-transcriptional processing arising from a poly(A) retrotransposon located close to the 3′ end of the gene (i.e., as reviewed in [25]). The Ensembl genome browser Boxer reference sequence (Broad CanFam2) indicates that the 5′ portion of the poly(A) retrotransposon is part of a putative exon in one of two predicted isoforms of the gene, although this annotation is absent in the latest build of the dog genome (CanFam 3.1) (Figure S3). If this sequence is part of an exon, then changes to the resulting protein due to altered exon structure may result in additional functional consequences. However, it should be noted that it is possible that aggressive behaviors represent an ancestral phenotype, so that the poly(A) expansion may be the wild-type and selective breeding has resulted in this being eliminated in most breeds other than the Malinois. Although the nature of additional *SLC6A3* variation does not suggest functional changes [12], [14], such effects should not be ruled out, and it may be difficult to clarify independent effects of variants. The complex polygenic nature of behavior and the range of behaviors associated with this insertion also predict that the insertion effects may be modified by additional genetic and environmental factors.

## Supporting Information

Figure S1 Partial pedigree information for dogs in our dataset.
(PDF)

Figure S2 Partial pedigree information for dogs in our database, noting dogs with at least one PolyA allele and owner reported at least one behavioral change.
(PDF)

Figure S3 Snapshot from Ensembl genome browser (CanFam2.0) indicating part of poly(A) retrotransposon as putative exon in Boxer reference sequence.
(PDF)

Table S1 Allele and genotype frequency across breeds, for Malinois versus Other breeds, and corresponding DAT-VNTR genotypes*.
(XLS)

Table S2 Dog information for subjects in behavioral analyses.
(XLS)

Table S3 Frequency distributions for owners responding “yes” to questions regarding dog glazing over, episodic aggression, and/or seizure.
(XLS)
