# Supplementary material for: Differences in Behavior and Activity Associated with a Poly(A) Expansion in the Dopamine Transporter in Belgian Malinois
Source: PLoS One. 2013 Dec 23;8(12):e82948. doi: 10.1371/journal.pone.0082948 (PMC3871558; doi:10.1371/journal.pone.0082948)

Supplemental Figure 2. Partial pedigree information for dogs in our dataset. Key: Orange square or circle: dog is included in our dataset, dog has at least one PolyA allele, and owner reported at least one behavioral change; Square: Male; Oval: Female; Dashed line: terminal parental node; Dashed diamond: gender not specified.

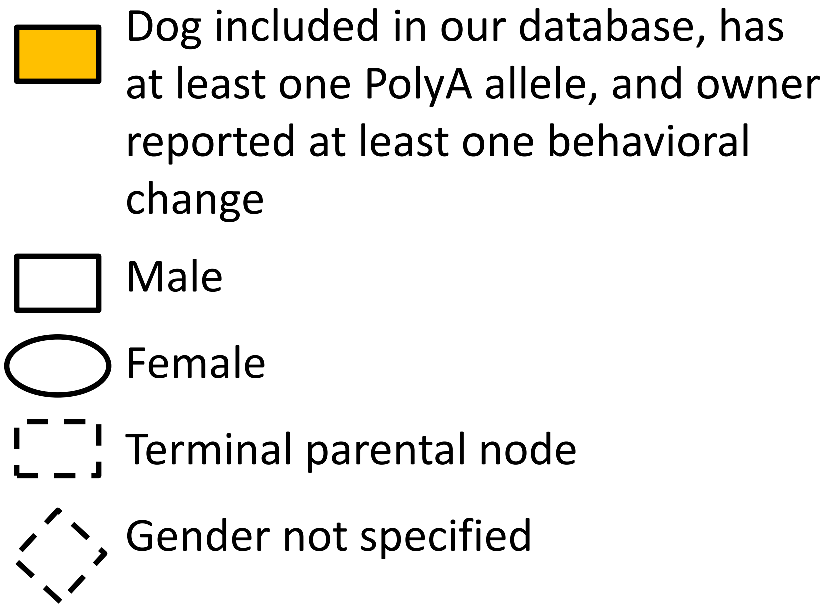

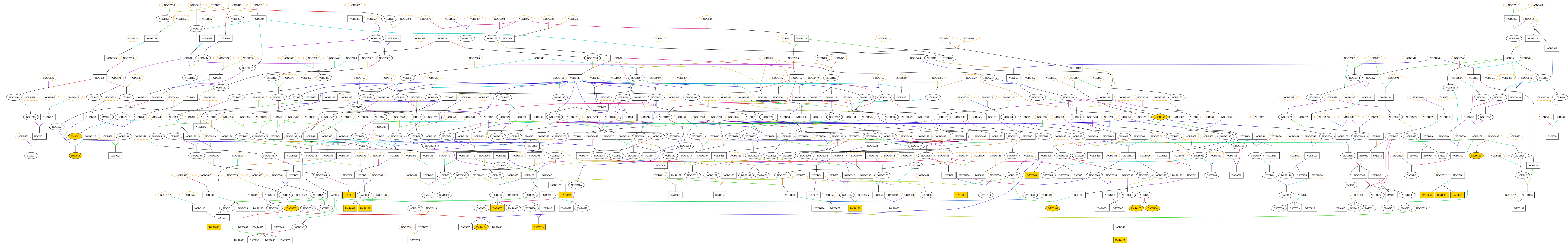

Supplement: Figure S2 — Partial pedigree information for dogs in our database, noting dogs with at least one PolyA allele and owner reported at least one behavioral change. (PDF) [file pone.0082948.s002.pdf]
