# Supplementary material for: Differences in Behavior and Activity Associated with a Poly(A) Expansion in the Dopamine Transporter in Belgian Malinois
Source: PLoS One. 2013 Dec 23;8(12):e82948. doi: 10.1371/journal.pone.0082948 (PMC3871558; doi:10.1371/journal.pone.0082948)

Supplemental Figure 3. Snapshot from Ensembl genome browser (CanFam 2.0) indicating part of poly(A) retrotransposon (red box) as putative exon in Boxer reference sequence. (http://dec2011.archive.ensembl.org/Canis_familiaris/Location/Compara_Alignments?chr=34;end=14249652;r=34:14249403-14249652;start=14249403).

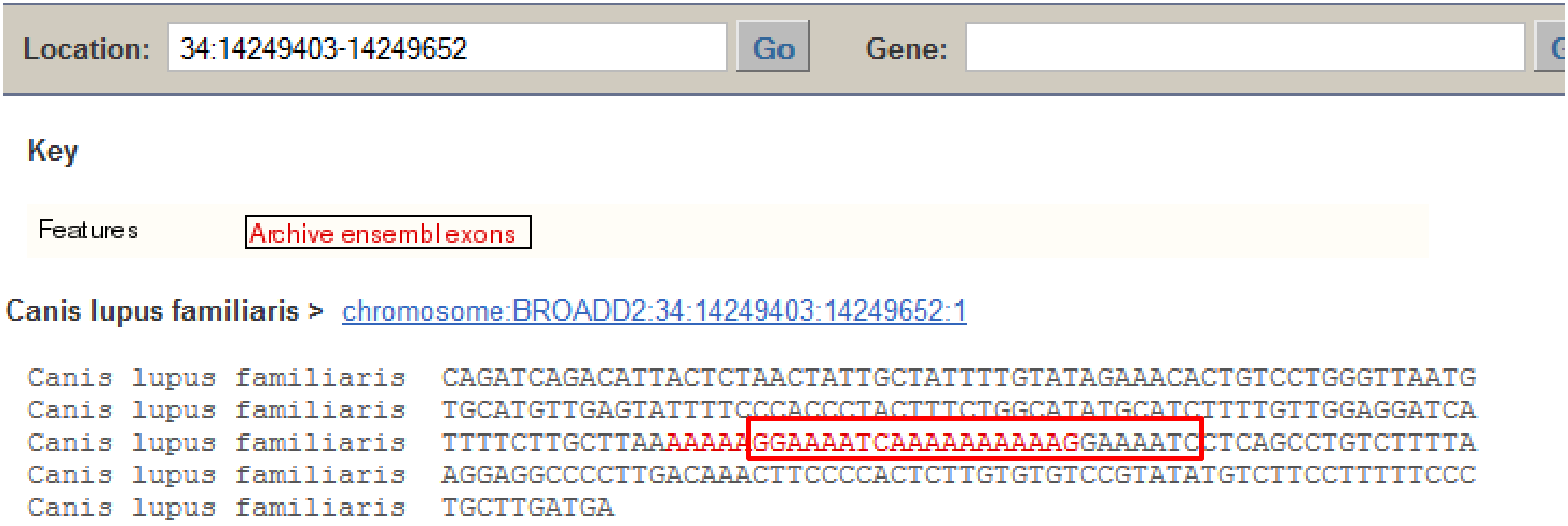

Supplement: Figure S3 — Snapshot from Ensembl genome browser (CanFam2.0) indicating part of poly(A) retrotransposon as putative exon in Boxer reference sequence. (PDF) [file pone.0082948.s003.pdf]
